# Supplementary material for: Proteomic Analysis of Salivary Secretions from the Tea Green Leafhopper, Empoasca flavescens Fabrecius
Source: Insects. 2024 Apr 22;15(4):296. doi: 10.3390/insects15040296 (PMC11050670; doi:10.3390/insects15040296)
Supplement: Supplementary file 1 [file insects-15-00296-s001.zip › insects-2922684-supplementary.pdf]

Supplemental Materials

Table S1. Primers of putative salivary secretory protein genes in RT-PCR.

Genes	Forward primer sequence (5′—3′)	Reverse primer sequence (5′—3′)
Efmucin1	ATGAACAATATTGAAGACCAACTTT	TTAACTCCTAACGGTGTTCTCCTTA
Efmucin2	ATGACGTCGCGATGGCTCGTGTT	GGGCTGTCCTGGTTGGCCGGGTTG
EfE3UPL	ATGCTGCCGCAAGGCTCTCACCTC	TCAGTCGTCGTCTGAGGCCACGTC
EfVDCC	ATGAGCGCCACCAACAACAACATCA	CTATAAGTGTGGGTCGTCCCCCT
EfP5CR	ATGTCTGCTGTTCTAGTGGAAC	TCATTTGCTGCCTAGTTCAA
EfelF3	ATGGAGCGTCTGCTGGTGGAGTGC	ACGGTCCCTGTCGTCGGCGGTG
EfTTP	ATGGCTTTGAAAGGTCCGAACCTG	TCAATCTATCGCAAGTTTTCTGA
EfCCDC	ATGAGGTTTTTTTGAGTCTAGCG	TTAAAGAGCTTTGACCTTCAGT
EfLRP	ATGAAACTGAGAAAGCACCTGCTC	ATGAGCAAACATTTCTAATTCAG
EfMME	ATGACCACCTCGCAAACAGTCATC	CTACCAGACTTGACATTTT
EfNUP	ATGAATCATATTTACATAGGG	TTATTTTGGTCGTCTCCTGAG
EfRGN	TCTTGGAGAAGGTCCTTA	CGCTTGTTGCTCCTTGGT
EfVg1	ATGGCGGCCATGTGGACCTCACTGC	GCCGGTGCTGTCCTGGCCGACAG
EfVg2	ATGGCTCAACCTTCTTCTTCTTC	TTAGGCCTTGCAGGACTCGGGC
EfOBP1	ATGACTTGCAAGCTGACCATCTT	TTAGTCCTTGAGGTGAATG
EfOBP2	ATGGTGACCAACATGGACAC	TCATGCTGAACCGAGGCCA
EfOBP3	ATGAACTCCTTCGTCGCCG	TGAGTGCGCGCTCCTCGAT
EfUP1	ATGTACCAATATAAGAGTAGTCTG	GGCTGGAACTGGCTGCGGGGC
EfUP2	ATGGGAACTGGCAGCAAAATGAAT	CTAGAACCTCTTGCACCTTGA
EfUP3	ATGGTTGTTGGAGCATTTCC	CTAAGAACAATTCTCACTTA

Table S2. Primers of putative salivary secretory protein genes in qRT-PCR.

Genes	Forward primer sequence (5′— 3′)	Reverse primer sequence (5′—3′)
Efmucin1	GTCAATACTTGCGGTGCCC	GCGTGGTCTTTGCTCGGT
Efmucin2	GCTCCTTTGTCGGACTCTACT	CTTGATTCCTGGGATGATTG
EfE3UPL	AAGCCATCAATGCTATCGG	GTCACTCTGTTCATCTGCCTCT
EfVDCC	GCTATCGCTGTAGACAACCTG	ACCCTCCATCACTTCCAACT
EfP5CR	ACTCGGGATGAAACCACAA	GCCCCAACCATGATAGGA
EfelF3	GGAGTGCGTGCGTCATAA	GTTCACCATTTGCTGCTTCT
EfTTP	CTGGTGCGTCAGTGTCCG	TCTTTCAGTCGTTGAGGGTATG
EfCCDC	CGGACTCGCAGTACACGC	GCCATATCTTTGCTCAGCCT
EfLRP	CTGCCTCGCCAACACTAAC	TTTCCAACCGACCTTTCG
EfMME	GAACTGGTGGGCTCCTGA	GCTTGATCCCTCCGTTATCT
EfNUP	TGATTGTAAAGCCAGCAGATAC	CGATTTTCCTTAGGTGGTGAG
EfRGN	TGAGGGAATGCTCTGGGTA	GCTTGTTGCTCCTTGGTGA
EfVg1	AGCGATGCCAGGAAACAC	TTGGGGAGGACAGACAGAAG
EfVg2	GTCGCTCCCTTGGTTGTG	CTCGTCATCGTCGTCTTCAT
EfOBP1	TCTGGTCGAGTGCATGTTTAG	CCTTGAGGTGAATGTTTTGC
EfOBP2	TGCAACTGATGATGAACTGTCA	CCACTTTCTTCGCCTTGACT
EfOBP3	CCGTCGTCCTGGCTCTAG	TTCCTTCGGGTGTCGTTG
EfUP1	GGACAACGCTCCTCCTACG	CCTGCAACGCATCCATCT
EfUP2	CCAAGCGTCAACATCATTCC	TCATTAACAGTTCCAGGCAAA
EfUP3	ATGTGGGTGATGAACTTGGG	CCTTTGCCGTTTCCTTGC
EfTubulin	GTGGTGCCAGGAGGTGACTT	ACCCTCTCCGACGTACCAGT
EfActin	ATGTGTGACGACGACGTAGCC	TTAGAAGCACTTCCTGTGGACG

Table S3. Summary of the proteins of the watery saliva of tea green leafhopper identified by LC-MS/MS.

Protein ID	Protein Name	No. of unique peptides	Protein domain	THMHH	TargetP	Signal P	Function group
A0A1B6EIE9	Eukaryotic translation initiation factor 3 subunit A	3	eIF-3 subunit A family	0	nucl	No	RNA binding
A0A1B6LSJ9	Uncharacterized protein	2		0	nucl	No	
A0A1B6M6J1	Gp_dh_N domain-containing protein	2	GAPDH, Gp_dh_N, NAD binding domain	0	cyto	No	Oxidoreductases
A0A1B6EU18	Uncharacterized protein	2	HSP70, heat shock protein 70 family	0	cyto	No	Heat shock protein
A0A1S5VZC4	Heat shock protein cognate 70-5	2	HSP70, heat shock protein 70 family	0	cyto	No	Heat shock protein
A0A1B6DRM1	Uncharacterized protein	2	InaF-motif	1	plas	No	
A0A482X3G0	Gp_dh_N domain-containing protein	2	GAPDH, Gp_dh_N, NAD binding domain	0	cyto	No	Oxidoreductases
A0A482WVW1	Uncharacterized protein	1		0	plas	Yes	
A0A1B6D700	Uncharacterized protein	1	RNA recognition motif. (a.k.a. RRM, RBD, or RNP domain)	0	nucl	No	RNA binding
A0A482X8K2	Uncharacterized protein	1	RMI1_N, SMN, UBA	0	cyto_nucl	No	RNA binding
A0A1B6HS38	Uncharacterized protein	1		0	nucl	No	
A0A482XN14	Uncharacterized protein	1	LUC7L2_1 protein	0	nucl	No	RNA binding
A0A1B6KNC8	Uncharacterized protein	1		0	extr	Yes	
A0A482X335	Uncharacterized protein	1		0	cyto_nucl	No	
A0A1B6FHX7	Uncharacterized protein	1		2	plas	Yes	
A0A1B6D9B6	Uncharacterized protein	1	Sodium channel and clathrin linker 1 (SCLT1/CAP-1A)	0	cyto	No	Ion transport protein
A0A1B6DWP9	Protein kinase domain-containing protein	1	Protein kinase	0	cyto	No	Ion transport protein
A0A482WFC3	Uncharacterized protein	1	Middle domain of eukaryotic initiation factor 4G (eIF4G), EIF-W2 protein domain	0	cyto_nucl	No	RNA binding
A0A482XT43	BTB domain-containing protein	1	Potassium channel tetramerisation domain, BTB/POZ domain	0	nucl	No	Ion transport protein
A0A1B6L8R3	Reverse transcriptase	1	reverse transcriptase (RT)	0	cyto	No	

	domain-containing protein						
A0A1B6C1M1	Mediator of RNA polymerase II transcription subunit 1	1	Mediator complex subunit 1 family	0	nucl	No	Transcriptional regulatory protein
A0A1B6CWA4	TPR_REGION domain-containing protein	1	Tetratricopeptide-like helical domain superfamily	0	cyto	No	protein binding
A0A1B6EK47	Rho-GAP domain-containing protein	1	Rho GTPase-activating protein domain	0	nucl	No	Signal transduction protein
A0A1B6F459	Elongation factor Tu	1	Elongation factor Tu GTP binding domain	0	mito	No	Transcriptional regulatory protein
A0A482XE38	Uncharacterized protein	1		0	extr	No	
A0A1B6D1L2	Uncharacterized protein	1	Mediator of CRAC channel activity, Orai-1	3	plas	No	
A0A1B6DRQ7	Uncharacterized protein	1		1	plas	No	
A0A482X4D7	RUN domain-containing protein	1	RUN domain superfamily	0	nucl	No	
A0A482XJ86	Uncharacterized protein	1	Integrin-alpha FG-GAP repeat-containing protein 2 (ITFG2)	0	cyto	No	
A0A1B6CYC8	Uncharacterized protein	1	Domain of unknown function (DUF4487)	0	plas	No	
A0A1B6DW74	Uncharacterized protein	1		0	mito	No	
A0A1B6IM19	Uncharacterized protein	1		0	cyto	No	
A0A482XGQ6	Uncharacterized protein	1	SART-1 family	0	nucl	No	Transcriptional regulatory protein
A0A1B6CKQ7	Uncharacterized protein	1	Zinc finger, RING/FYVE/PHD-type	0	cyto	No	DNA binding
A0A1B6CUK3	Pescadillo homolog	1	Regulator of Ty1 transposition protein 107, BRCT domain	0	cyto	No	RNA binding
A0A1B6CY97	Dolichyl-diphosphooligosaccharid e--protein glycosyltransferase subunit 2	1	Oligosaccharyltransferase subunit Ribophorin II	3	plas	Yes	Protein transporter
A0A1B6DX26	Sec3-PIP2_bind domain-containing protein	1	Exocyst complex component Sec3, PIP2-binding N-terminal domain	0	cyto	No	Vacuolar transporter
A0A1B6EVI0	Uncharacterized protein	1	Zinc finger C2H2-type	0	nucl	No	nucleic acid binding
A0A1B6EXC8	Proteasome subunit beta	1	Proteasome beta-type subunit, conserved site	0	cyto	No	Peptidases
A0A1B6FN75	Uncharacterized protein	1	HSP60, heat shock protein 60 family	0	cyto	No	Heat shock protein

A0A1B6F247	FERM domain-containing protein	1	FERM domain (F for 4.1 protein, E for ezrin, R for radixin and M for moesin)	0	mito	No	molecular biology
A0A1B6FUL5	Ubiquitin-like domain-containing protein	1	Ubiquitin family	n0	cyto	No	Ubiquitin
A0A1B6GHV6	Uncharacterized protein	1	HSP60, heat shock protein 60 family	0	cyto	No	Heat shock protei
A0A1B6IZ35	Uncharacterized protein	1		0	cyto_nucl	No	
A0A1B6K4K8	Uncharacterized protein	1	Neurotransmitter-gated ion-channel ligand binding domain	4	plas	Yes	Ion transport protein
A0A1B6KEM3	Uncharacterized protein	1	Carnitine deficiency-associated protein 3	0	cyto	No	Unknown
A0A1B6KUR1	PEHE domain-containing protein	1	PEHE domain	0	nucl	No	Transcriptional regulatory protein
A0A1B6KWK7	H15 domain-containing protein	1	Linker histone H1/H5, domain H15	0	nucl	No	DNA binding
A0A482WRC5	Uncharacterized protein	1	Kinesin motor domain superfamily	0	nucl	No	Cytoskeleton protein
A0A482XHM6	Uncharacterized protein	1		0	mito	Yes	
A0A482XJ52	Uncharacterized protein	1	Signal peptide, PRO_5019777043	0	extr	Yes	Signal peptide
A0A0A1GY74	Vitellogenin	1	Vitellogenin#N-terminal lipid transport domain	0	extr	Yes	lipid transporter
A0A125RA07	Actin	1	Actin	0	cysk	No	Cytoskeleton protein
A0A1B6C224	RRM domain-containing protein	1	RNA recognition motif domain	0	nucl	No	RNA binding
A0A1B6C2K7	Uncharacterized protein	1	Endonuclease/exonuclease/phosphatas e	0	nucl	No	nucleic acid binding
A0A1B6CBL8	Uncharacterized protein	1	Signal peptide, PRO_5008580268	0	extr	Yes	Signal peptide
A0A1B6CIJ1	Integrase_H2C2 domain-containing protein	1	Integrase zinc-binding domain	0	mito	No	nucleic acid binding
A0A1B6CHQ6	Uncharacterized protein	1	ABC transporter, ATP-Binding Cassette (ABC) superfamily	0	cyto	No	ABC transporter
A0A1B6CRT5	Uncharacterized protein	1	Helicase superfamily 1/2, ATP-binding domain	0	cyto	No	Transcriptional regulatory protein
A0A1B6CV32	Uncharacterized protein	1	Cys/Met metabolism PLP-dependent enzyme family	0	cyto	No	Transferases
A0A1B6CV99	Uncharacterized protein	1	Protein of unknown function	0	extr	Yes	Signal peptide
A0A1B6CX30	RAWUL domain-containing protein	1	RAWUL domain RING finger- and WD40-associated ubiquitin-like	0	nucl	No	Ubiquitin

A0A1B6D4A8	Uncharacterized protein	1		0	cyto_nucl	No	
A0A1B6D6U9	Uncharacterized protein	1		0	nucl	No	
A0A1B6D6X4	Uncharacterized protein	1	S-adenosyl-L-methionine-dependent methyltransferase	0	plas	Yes	Transferases
A0A1B6D8E8	Pyrroline-5-carboxylate reductase	1	Pyrroline-5-carboxylate reductase, dimerisation domain	0	cyto	Yes	Oxidoreductase
A0A1B6DBC7	Uncharacterized protein	1	High mobility group box domain superfamily	0	mito	No	DNA binding
A0A1B6DEE5	DNA ligase	1	DNA ligase, ATP-dependent	0	nucl	No	Ligase
A0A1B6DK89	BZIP domain-containing protein	1	Basic Leucine Zipper Domain (bZIP domain)	0	nucl	No	
A0A1B6DP13	Ribosomal_L16 domain-containing protein	1	Ribosomal protein L16p/L10e	0	nucl	No	Ribosomal protein
A0A1B6E485	IPPc domain-containing protein	1	Inositol polyphosphate-related phosphatase	0	cyto	No	phosphatase
A0A1B6EBZ4	Uncharacterized protein	1	Zinc finger, CXXC-type	0	nucl	No	nucleic acid binding
A0A1B6ENL3	Cyclic nucleotide-binding domain-containing protein	1	Cyclic nucleotide-binding domain	0	nucl	No	nucleic acid binding
A0A1B6EWL3	SAP domain-containing protein	1	SAP domain	0	nucl	No	Transcriptional regulatory protein
A0A1B6F0Q0	HTH psq-type domain-containing protein	1	helix-turn-helix (HTH) psq-type domain	0	cyto	No	DNA binding
A0A1B6F210	Uncharacterized protein	1	HSP60, heat shock protein 60 family	0	mito	No	Heat shock protein
A0A1B6F2T3	OB_NTP_bind domain-containing protein	1	Oligonucleotide/oligosaccharide-binding (OB)-fold	0	extr	No	nucleic acid binding
A0A1B6FGQ7	RING-type domain-containing protein	1	Zinc finger, RING-type	0	nucl	No	nucleic acid binding
A0A1B6FKR6	Uncharacterized protein	1		0	nucl	No	
A0A1B6FMQ0	Uncharacterized protein	1	Six-bladed beta-propeller, TolB-like	1	plas	Yes	Transmembrane protein
A0A1B6FUZ7	Uncharacterized protein	1		0	plas	No	
A0A1B6FX17	Uncharacterized protein	1	Putative sperm flagellar membrane protein	1	plas	No	Transmembrane
A0A1B6G3M1	Uncharacterized protein	1	Helicase superfamily 1/2, ATP-binding	0	cyto	No	Helicase

			domain				
A0A1B6G9X0	Uncharacterized protein	1		0	mito	No	
A0A1B6GB27	Carboxylic ester hydrolase	1	Carboxylesterase, type B	0	plas	No	hydrolase
A0A1B6GEQ5	Uncharacterized protein	1		0	nucl	No	
A0A1B6GW14	Uncharacterized protein	1	AH/BAR domain superfamily	0	nucl	No	Protein binding
A0A1B6GYZ2	Uncharacterized protein	1	Centriolar coiled-coil protein of 110kDa	0	nucl	No	Cytoskeleton
A0A1B6H242	Uncharacterized protein	1		0	cyto_nucl	No	
A0A1B6H6M2	Uncharacterized protein	1	Short-chain dehydrogenase/reductase SDR	0	cyto	No	Oxidoreductase
A0A1B6HHN6	Uncharacterized protein	1	AT hook, DNA-binding motif	0	nucl	No	DNA binding
A0A1B6HI94	Uncharacterized protein	1		0	nucl	No	
A0A1B6HIM1	Uncharacterized protein	1	E3 ubiquitin-protein ligase, SMURF1 type	0	cyto	No	ubiquitin protein ligase
A0A1B6HJQ6	Rap-GAP domain-containing protein	1	Rap GTPase activating protein domain	0	cyto	No	Signal transduction protein
A0A1B6HRD0	Uncharacterized protein	1		0	nucl	No	
A0A1B6HSN9	Uncharacterized protein	1	non-motor microtubule binding protein	0	cyto_nucl	No	microtubule binding
A0A1B6HTU9	Uncharacterized protein	1		0	nucl	No	
A0A1B6HUL5	Uncharacterized protein	1	Zinc finger C2H2-type	0	nucl	No	nucleic acid binding
A0A1B6HVU0	Uncharacterized protein	1		0	mito	No	
A0A1B6HXS5	Protein kinase domain-containing protein	1	Protein kinase domain	0	cyto	No	protein kinase
A0A1B6I3R8	Uncharacterized protein	1		0	nucl	No	
A0A1B6I701	VWFC domain-containing protein	1	VWFC domain	0	nucl	No	protein binding
A0A1B6IEA4	Uncharacterized protein	1	Signal peptide, PRO_5008584999	0	extr	Yes	Signal peptide
A0A1B6IIU7	Uncharacterized protein	1		0	nucl	No	
A0A1B6IYH7	Uncharacterized protein	1		0	nucl	No	
A0A1B6J5Y8	Uncharacterized protein	1		0	mito	No	
A0A1B6J6A8	Uncharacterized protein	1		0	nucl	No	
A0A1B6JQC6	Uncharacterized protein	1	Zinc finger C2H2-type	0	nucl	No	nucleic acid binding
A0A1B6KM25	Uncharacterized protein	1		0	cyto	No	
A0A1B6KWT4	ASD2 domain-containing protein	1	Apx/Shroom domain ASD2	0	nucl	No	Cytoskeleton protein

A0A1B6L311	RRM domain-containing protein	1	RNA recognition motif. (a.k.a. RRM, RBD, or RNP domain)	0	nucl	No	RNA binding
A0A1B6L6P4	Uncharacterized protein	1		0	cyto	No	
A0A1B6LH55	Uncharacterized protein	1		0	cyto	No	
A0A1B6LPT6	Dimer_Tnp_hAT domain-containing protein	1	hAT family C-terminal dimerisation region	0	cyto	No	Nucleotide metabolism and transport
A0A1B6LXQ0	Uncharacterized protein	1		0	extr	No	
A0A1B6MLT4	Uncharacterized protein	1	Peptidase M13, domain 2	1	E.R.	Yes	metalloendopeptidas e
A0A1B6MSI5	Uncharacterized protein	1	Heat shock protein Hsp90 family	0	cyto	No	Heat shock protein
A0A220XK81	Hepatocyte nuclear factor 4	1	nuclear hormone receptor family	0	nucl	No	DNA binding
A0A482WE63	FACT complex subunit SSRP1	1	High mobility group (HMG) box domains	0	nucl	No	DNA binding
A0A482WH06	PH domain-containing protein	1	Pleckstrin homology domain	0	nucl	No	
A0A482WJK3	Uncharacterized protein	1	BRCT domain superfamily	0	cyto	No	intracellular signal transduction
A0A482WJW3	Tet_JBP domain-containing protein	1	Oxygenase domain of the 2OGFeDO superfamily	0	nucl	No	Oxidoreductase
A0A482WJY4	DNA-directed RNA polymerase III subunit RPC6	1	eukaryotic RPC34/RPC39 RNA polymerase subunit family	0	cyto	No	
A0A482WPY7	Uncharacterized protein	1	EF-hand domain pair	1	cyto	No	
A0A482WQ76	Uncharacterized protein	1		1	extr	Yes	
A0A482WWR9	Uncharacterized protein	1	Signal peptide, PRO_5019819340	0	extr	Yes	Signal peptide
A0A482WXR1	Uncharacterized protein	1		0	cyto	No	
A0A482WZC8	Uncharacterized protein	1	Protein transport Sec61-beta/Sbh	1	nucl	No	protein transport
A0A482X2I5	Uncharacterized protein	1	Tetratricopeptide-like helical domain superfamily	0	nucl	No	Protein interaction
A0A482X370	Uncharacterized protein	1	OST-HTH/LOTUS domain	0	nucl	No	RNA binding
A0A482X7M5	Anaphase-promoting complex subunit 10	1	APC10 family, multi-subunit E3 protein ubiquitin ligase	0	cyto	No	
A0A482X7R4	G_PROTEIN_RECEP_F1_2 domain-containing protein	1	G-protein coupled receptor 1 family	7	plas	No	
A0A482X7Z5	Uncharacterized protein	1		0	nucl	No	

A0A482X821	Uncharacterized protein	1	TRAFAC class myosin-kinesin ATPase superfamily	0	nucl	No	microtubule binding
A0A482XA40	Uncharacterized protein	1	eIF4E, Eukaryotic translation initiation factor 4E binding protein	0	nucl	No	transporter protein
A0A482XAJ9	Uncharacterized protein	1	Optic atrophy 3-like	0	E.R._mito	Yes	unknown
A0A482XAR4	Uncharacterized protein	1		0	mito	No	
A0A482XBD9	Uncharacterized protein	1		0	nucl	No	
A0A482XCB9	SAM domain-containing protein	1	Sterile alpha motif/pointed domain superfamily	1	cyto	No	
A0A482XEC0	Uncharacterized protein	1	SH3-like domain, Sterile alpha motif/pointed domain superfamily	0	nucl	No	
A0A482XFS4	Uncharacterized protein	1	AMP-dependent synthetase/ligase	0	cyto	No	
A0A482XGW6	Uncharacterized protein	1	Zinc finger C2H2-type	0	nucl	No	nucleic acid binding
A0A482XJS4	Uncharacterized protein	1		0	cyto	No	
A0A482XKF3	Ribosomal_L14e domain-containing protein	1	Ribosomal protein L14e domain	0	nucl	No	Translation
A0A482XL42	N-acetyltransferase domain-containing protein	1	Acyl-CoA N-acyltransferases (Nat）	0	mito	No	Transferases
A0A482XMX7	Uncharacterized protein	1		0	nucl	No	
A0A482XN76	Uncharacterized protein	1	K Homology domain, type 1 superfamily	0	cyto	No	RNA binding
A0A482XQH2	Uncharacterized protein	1		0	nucl	No	
A0A482XSJ7	Uncharacterized protein	1	Death-like domain superfamily	0	cyto	No	Cell cycle, Apoptosis
B6E9U3	Odorant binding protein 3	1	Pheromone/general odorant binding protein superfamily	0	extr	Yes	odorant binding
G1C2J1	Tubulin alpha chain	1	Tubulin/FtsZ, GTPase domain superfamily	0	cysk	No	Cytoskeleton protein
J9ZWA5	Histone H2A	1	C-terminus of histone H2A	0	nucl	No	

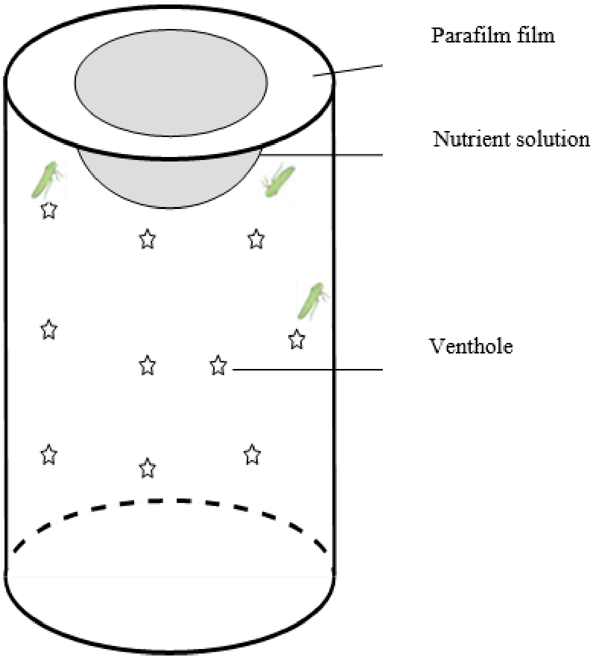

Figure S1. Schematic diagram of leafhopper saliva collection device (Pan et al., 2021) [28]. Each saliva collection container was placed at one side of sterile glass tube (5.5 cm ×10 cm), and add a layer of Parafilm on the outermost side to prevent leakage, while the opposite end was used to introduce the leafhoppers. After leafhopper introduction, the open end of the glass tube was secured with a piece of cheesecloth and a rubber band. The glass tubes were transferred to chambers maintained at 25 ± 2 °C and 70 ± 5% relative humidity, under 14:10 L:D photoperiod.
